# Recommendations for improving the end-of-life care system for homeless populations: A qualitative study of the views of Canadian health and social services professionals

**DOI:** 10.1186/1472-684X-11-14

**Published:** 2012-09-15

**Authors:** Ryan McNeil, Manal Guirguis-Younger, Laura B Dilley

**Affiliations:** 1 British Columbia Centre for Excellence in HIV/AIDS, Vancouver, British Columbia, Canada; 2 Faculty of Human Sciences, Saint Paul University, Ottawa, ON, Canada; 3 Faculty of Education, Simon Fraser University, Surrey, BC, Canada

## Abstract

**Background:**

Homeless populations have complex and diverse end-of-life care needs. However, they typically die outside of the end-of-life care system. To date, few studies have explored barriers to the end-of-life care system for homeless populations. This qualitative study involving health and social services professionals from across Canada sought to identify barriers to the end-of-life care system for homeless populations and generate recommendations to improve their access to end-of-life care.

**Methods:**

Semi-structured qualitative interviews were conducted with 54 health and social services professionals involved in end-of-life care services delivery to homeless persons in six Canadian cities (Halifax, Hamilton, Ottawa, Thunder Bay, Toronto and Winnipeg). Participants included health administrators, physicians, nurses, social workers, harm reduction specialists, and outreach workers. Interviews were audio-recorded, transcribed verbatim and analysed thematically.

**Results:**

Participants identified key barriers to end-of-life care services for homeless persons, including: (1) insufficient availability of end-of-life care services; (2) exclusionary operating procedures; and, (3) poor continuity of care. Participants identified recommendations that they felt had the potential to minimize these barriers, including: (1) adopting low-threshold strategies (e.g. flexible behavioural policies and harm reduction strategies); (2) linking with population-specific health and social care providers (e.g. emergency shelters); and, (3) strengthening population-specific training.

**Conclusions:**

Homeless persons may be underserved by the end-of-life care system as a result of barriers that they face to accessing end-of-life care services. Changes in the rules and regulations that reflect the health needs and circumstances of homeless persons and measures to improve continuity of care have the potential to increase equity in the end-of-life care system for this underserved population.

## Background

Tens of thousands of people in North America experience homelessness every year [1,2]—that is, live in conditions unfit for human habitation or temporary or emergency accommodations without housing alternatives [3]—and many thousands more are at risk of homelessness at any given time [1,2]. Homeless populations in North America, while highly heterogeneous in terms of age, gender, sexuality, and ethnicity, suffer disproportionately from poor health in comparison to housed populations [4]. Chronic health conditions that are common among the homeless include chronic lung diseases [5], circulatory diseases [6], and diabetes [7]. Homeless persons also experience higher incidences of substance use [8,9], severe mental illness [10,11], and infectious diseases such as HIV/AIDS [12,13] and Hepatitis C [14]. Daily challenges associated with homelessness (e.g. food insufficiency, exposure, etc.) [4,15,16] and barriers to accessing health care services (e.g. discrimination, lack of insurance, etc.) [4,17,18] make it difficult to manage medical needs, leading to further deteriorations in overall health. Homeless populations subsequently have among the highest all-cause mortality rates of any population in North America [19-23].

Homeless persons have a high level of need for end-of-life care services [24,25] and these needs may be increasing due to the steady growth in the number of homeless older adults [26,27]. It is estimated that more than 58,000 seniors (i.e. 62 years or older) will experience homelessness annually in the US by 2020 [26] and, while estimates are not available for Canada, researchers in various cities have observed upward trends [27]. High levels of morbidity among homeless older adults [28], in combination with the natural progression of health challenges common among this population (e.g., HIV/AIDS, HCV, etc.), suggest that the end-of-life care system will likely see an increased demand for its services among the homeless in the immediate future.

While the demand for end-of-life care services may be growing among the homeless in North America, this population faces many barriers to accessing end-of-life care services [24,25,29,30]. In North America, the end-of-life care system is largely premised on a series of assumptions that do not reflect the experiences and circumstances of homeless populations. Specifically, the end-of-life care system generally assumes that prospective clients are housed, supported by family and friends, and able to pay for supplementary care. In Canada, where our research was conducted, hospice and palliative care services are underdeveloped [31] and are structured in ways that limit access for homeless populations. For example, existing service structures emphasize family caregivers and dying-in-place (i.e., the home) [31,32]. Accordingly, in many regions, end-of-life care services are oriented toward providing home care support and potentially limit access for homeless or precariously housed persons. Hospice and hospital-based end-of-life care services are also available to provide an additional source of care in many communities, especially in urban centres [31]. However, homeless populations are often unable to access hospice or hospital-based end-of-life care due to rules and regulations (e.g. anti-drug policies, codes of behaviour, etc.) that exclude substance-using populations [29,30]. For example, research suggests that, among those who continue to use alcohol or illicit drugs at end-of-life, abstinence-only policies and discrimination within hospice and palliative care settings serve as a significant barrier to care [29,30]. Furthermore, homeless persons who use alcohol or drugs may also refuse referrals to hospice and palliative care services due to a range of factors, including real or perceived discrimination in these settings or the preference to die in a familiar environment (e.g., emergency shelter, hostel, etc.) [30].

Due to these barriers, homeless persons often die with poor health care support [33] and without accessing the end-of-life care system [34]. Song et al. [35] have reported that these barriers may serve as a source of anxiety among homeless persons—namely, that they might have poor access to necessary care (e.g., pain and symptom management) due to financial barriers. Several ways to improve end-of-life care services delivery to homeless populations have been previously identified, and have included individual-level and environmental recommendations. For example, researchers have recommended that advance care planning be undertaken with homeless persons and noted that this population is willing to document its end-of-life care preferences [35-39]. Researchers have also documented the benefits of emergency shelter-based end-of-life care services delivery, including cost savings [24] and cultural competence [24,30,40]. And yet, research has not been conducted that has explored ways to improve the end-of-life care system as a whole for homeless populations. Research providing systems-level recommendations is urgently needed to identify structural changes that have the potential to increase access and equity in end-of-life care services for homeless populations.

This article presents recommendations for improving the end-of-life care system for homeless persons based on research conducted in six Canadian cities as part of a national study of homelessness and end-of-life care. The main objectives of this study were to identify barriers to end-of-life care services delivery to homeless persons and identify recommendations to improve the end-of-life care system for this population. The findings presented here take into account the perspectives of health and social services professionals providing care to homeless persons at end-of-life. While this study was carried out in a country with universal health insurance, our findings provide insights that may strengthen end-of-life care services delivery to homeless persons elsewhere given the barriers they face to accessing care even when healthcare coverage is available [41].

## Methods

### Study design and participants

We conducted qualitative interviews with health and social services professionals in six Canadian cities between February 2007 to August 2008 in which we explored the social and structural factors that impact end-of-life care services delivery to homeless persons. In particular, we aimed to identify barriers to providing end-of-life care services to this population, as well as identify recommendations improve equity in the end-of-life care system. We invited health and social services professionals involved in end-of-life care services delivery to homeless persons in Halifax, Ottawa, Hamilton, Toronto, Thunder Bay, and Winnipeg to participate in this study. An advisory committee comprised of regional experts (i.e., senior health and social services administrators for homeless service organizations), as well as existing relationships with homeless service organizations in Ottawa and Toronto, helped us to identify key informants in those cities. We identified key informants in the remaining cities by conducting a scoping review of health services for homeless persons in those cities.

Letters or emails were sent to seventy-three potential participants to provide them with information about the study and invite them to participate. Fifty-four individuals (74%) agreed to participate, representing a range of professional roles, including physicians (6), nurse practitioners (2), nurses (16), social workers (5), emergency shelter or supportive housing executive directors and/or senior managers (9), harm reduction specialists (5), outreach workers (7), and personal support workers (4). Fifteen participants worked in low-threshold hospices (i.e. allowing onsite alcohol use, providing clean syringes, and permitting off-site illicit drug use) and the remainder of participants worked primarily in other community settings where they provided care to homeless persons unable to access the end-of-life care system. A minority of participants (6) worked in both hospital and community settings but reported that they seldom provided end-of-life care to this population in hospital.

### Data collection

Semi-structured qualitative interviews were conducted with participants at their workplace or alternate location of their choosing. The majority of interviews were conducted by the lead author (RM), a qualitative health researcher, while the remaining interviews were conducted by the study principal investigator (MGY), a clinical psychologist. The interview guide focused on the end-of-life care needs of homeless persons and barriers and facilitators to providing end-of-life care to this population. Participants identified few facilitators and instead tended to make recommendations to improve care. Participants were asked throughout the interview to identify strategies to improve the end-of-life care system for homeless persons. An abridged version of this interview guide is provided in Table 1. Interviews were audio recorded and ranges in length from 45 to 120 minutes, although the majority were approximately 60 minutes in length. Interviews were transcribed verbatim by research assistants and we conducted a data fidelity check by reviewing transcripts while simultaneously listening to the audio files.

**Table 1:** Examples of interview questions

**Topic Area: Barriers to accessing the end-of-life care system**	
*Questions*	*Probes*
What do you feel prevents homeless persons from receiving referrals to palliative care services?	*Why do you feel they are not referred to:*
	▪ *Hospice care?*
	▪ *Hospital-based care?*
What do you feel prevents homeless persons from receiving referrals to palliative care services?	*How do you feel their access is limited by:*
	▪ *Real or perceived discrimination?*
	*Alcohol or illicit drug use?*
What do you feel are the challenges to palliative care services delivery to homeless persons?	*How is palliative care services delivery to homeless persons adversely affected by:*
	▪ *Alcohol or illicit drug use?*
	▪ *Mental illness?*
**Topic Area: Facilitators to accessing the end-of-life care system**	
*Questions*	*Probes*
What do you feel facilitates access to palliative care services for homeless persons?	*What makes it easier for them to access palliative care services?*
What do you do to help homeless people access palliative care services?	*Can you describe when you helped someone access palliative care services?*
	*How did you help them?*
**Probes: Ask throughout as appropriate**	
*Questions*	
What changes do you feel would minimize the impact of [name of barrier]?	
What types of programs do you feel would minimize the impact of [name of barrier]?	

### Data analysis

The goal of analysis for this article was to identify perceived barriers to the end-of-life care system for homeless persons, as well as recommendations to improve services for this population. We imported the transcripts into NVivo qualitative data analysis software (version 8) to facilitate coding. A preliminary set of three categories (e.g. access to end-of-life care, community partnerships, and education and training) was extracted from lead author’s field notes and used to provide an initial framework for the analysis. Two of us (RM & LBD) independently coded the data by drawing on constant comparison methods, wherein preliminary categories were revised and emerging categories were identified and expanded through constant comparison to the data [42,43]. We regularly met to discuss emerging categories, with any revisions to the coding framework made by consensus. All authors discussed emerging themes to aid in framing the findings in relation to existing literature. Once the final categories were established, one of us (RM) re-coded sections of the data to ensure the credibility of these categories.

### Ethics

This study was approved by the research ethics committees at the University of British Columbia and Saint Paul University. Informed consent was obtained prior to interviews and participants retained a duplicate copy of the informed consent protocol.

## Results

Participants identified key barriers to end-of-life care services for homeless persons and recommendations for improving the end-of-life care system for this population. Five themes are organized into two domains: first, barriers to end-of-life care services; and, second, recommendations to improve the end-of-life care system. Barriers to and recommendations for improving the end-of-life care system were consistent across the cities included in this study, although the availability of low threshold services in two cities (Ottawa and Toronto) was perceived to minimize some barriers to care. Where participants are quoted directly, they are identified by profession to provide insight into the type of support they provide. Organizations named by participants have been replaced with generic descriptions to preserve their anonymity.

### Perceived barriers to the end-of-life care system

#### Availability of end-of-life services and supports

Participants noted that, although end-of-life care services struggled to meet local demand, what services were available were generally inaccessible to homeless populations. Participants noted that homeless populations were unable to access end-of-life care services as a result of a lack of caregiver support and/or financial resources. Participants reported that end-of-life care services in their communities assumed that clients were stably housed and supported by caregivers or had the financial resources to pay for care (e.g. assisted living facilities). As a consequence, they felt that their clients were unable to access these services. For example:

"[The emergency shelter] has guys who are seniors and they have not been accepted in retirement or nursing home care [the primary palliative care providers in the community]. They would qualify except for behavioural issues and they have no family support. I don’t know what the issues are but they obviously have no money to pay to get in. They have no family to advocate [for them]. (Emergency Shelter Director)"

"We kind of rightly or wrongly think palliative care and hospices are for the middle class. We never think about the poor. We are assuming the poor will automatically get in but because there is often a cost component, sometimes the homeless are left to die on the streets. (Emergency Shelter Director)"

#### Operating policies that exclude homeless populations

Participants noted that end-of-life care providers in their communities had largely adopted operating policies that excluded homeless populations from accessing services (e.g., anti-drug policies, codes of conduct, etc.). Participants felt that these operating policies privileged ‘normative patients’ (e.g., persons who were housed, had family, and conformed to procedures) and excluded homeless persons on the basis of a range of conditions common among this population (e.g., mental illness and substance use). In particular, anti-drug policies were identified as a barrier to care and, where formal policies did not exist, participants reported that substance-using homeless persons were identified by intake personnel as disruptive and, on the basis of this, denied services. Participant accounts suggest that these operating policies were perceived as discriminatory because they prevented a particular population (e.g., homeless persons) from accessing services, thus reinforcing inequities in access to the end-of-life care system. As two participants noted:

"It’s driven by the fact that the health care system has failed that population…When they are trying to access care in the mainstream facility, they experience discrimination and disrespect and poor care. (Nurse Practitioner)"

"For some people, it is addictions or mental illness that prevents them from getting the best care. It’s the attitude of their health care provider not being particularly welcoming or understanding of their situation…I think they’re certainly stereotyped in a negative way and I think that people are kind of inclined to say “Oh the homeless, that homeless guy” and it just conjures up a whole set of connotations about how we expect them to look, how we expect him to act and how we can treat them. (Physician)"

#### Lack of continuity of care

Participants expressed frustration with the lack of continuity of care for this population. They highlighted two particular challenges with implications for the end-of-life care system. First, participants noted that poor continuity of care (e.g. lack of follow-up, poor discharge planning, etc.) often precipitated the need for end-of-life care services among homeless persons with chronic diseases (e.g. HIV/AIDS). For example:

"It is a real challenge in those patients where the failure of the system or their inability to comply with therapy has resulted in a much worse outcome that would have otherwise been the case… I don’t know if an earlier diagnosis would have made a difference for the patient who sticks in my mind… Maybe it would have, maybe it wouldn’t have. I think that it is those kinds of cases that are more salient in my mind. (Physician)"

Second, participants noted that poor discharge planning placed an undue burden on community agencies ill-equipped to provide end-of-life care services. Participants reported that homeless persons were frequently discharged directly to emergency shelters even though these settings could provide only limited care to clients with complex medical needs. For example:

"There is no discharge planning for this population. They are pushed out of the hospitals to make room for beds because there are bed shortages. They do very little in the way of planning to discharge homeless people. They are easily shoved to the street or shelter…You have incidents where you are having people dropped off [at the emergency shelter] by ambulance. Over the last month, they have been a little bit more courteous in calling. I think that has to do with the recent death of this fellow but I won’t bet that it will continue because there doesn’t seem to be a lot of continuity there. (Shelter director)"

### Participant recommendations to improve the end-of-life care system

#### Low-threshold strategies

Participants strongly recommended that the end-of-life care system adopt low-threshold approaches, which have minimal requirements for admission and care. Participants emphasized that conventional approaches requiring drug or alcohol abstinence restricted access to end-of-life care services for substance-using homeless populations. Participants reported that it was important that end-of-life care providers acknowledge that changes to rules and regulations were needed for the purpose of serving this population. Some participants noted that integrating harm reduction strategies for alcohol use (i.e. prescribing alcohol and managing intake) and illicit drug use (i.e. providing clean needles and permitting off-site illicit drug use) were low-threshold strategies with the potential to improve end-of-life care services for this population. These participants observed that, although

Furthermore, many participants articulated that this adoption of harm reduction strategies expressed a commitment to serving homeless persons and awareness of this population’s life circumstances. For example:

"People died outside on the streets because [end-of-life care providers] couldn’t provide that. We agreed to walk outside on the street with these people. [Harm reduction] is part of walking down the road, so that they don’t go out and drink Listerine. (Emergency shelter director)"

#### Partnering community agencies with the end-of-life care system

Participants strongly believed that the wide variety of health and social services agencies accessed by homeless persons (e.g. shelters, soup kitchens, syringe exchange programs, etc.) should be formally partnered with the end-of-life care system. Participants articulated how the trust developed between these agencies and homeless populations helped to mediate access to a range of other services (e.g., primary care, specialists, etc.) and could perform a similar function in the context of end-of-life care. Furthermore, participants reported that these agencies, and especially trusted staff, were able to monitor changes in health status over time due to their sustained contact with this population and mediate access to health and end-of-life care services. For example:

"We work together at three sites. Because many of our patients that we have [in the hospice] have been known to the other two sites, there's kind of a family. In that way, we help each other and we communicate with each other. As far as other facilities go, we use what's out there in the community. Our patients may be known to some community health centers. (Nurse)"

Participants felt that, where partnerships were weak or did not exist, they needed to be developed. Several participants also noted that third-party advocates (e.g., patient navigators) could play a role in acting as liaisons between community agencies and the end-of-life care system to strengthen these partnerships. For example:

It would be helpful to have like individuals who serve as bridges between the [health and social services] systems. Usually, people don’t want a system. They want a person that they can call so, the doctor or the health care team in the hospital would prefer that there is a person that they can call to help them out rather than saying “These are the steps that you do.” I think that people are the key to building bridges. (Physician)

#### Strengthening training for end-of-life care professionals

Participants reported that increased training was needed to strengthen the capacity of healthcare professionals to address the complex and diverse needs of homeless populations at end-of-life (e.g. pain and symptom management, substance use, etc.). Participants noted that, while they valued the clinical expertise of healthcare professionals, clinicians often lacked experience in areas such as mental health and substance use that limited their effectiveness and openness to best practices. One participant remarked:

"When you’re trained in your profession, you’re trained in a certain way. If harm reduction wasn’t in your training, you’re not going to know anything about it. How can you expect somebody to embrace that with open arms if they know nothing about it? (Harm Reduction Specialist)"

Participants acknowledged that they needed to strengthen their training in these areas, as well as provide training opportunities for students. Participants emphasized that community-academic partnerships had the potential to strengthen the capacity of healthcare professionals to provide care to homeless populations, while potentially decreasing discrimination towards homeless populations. As one participant reported:

"The unwelcomeness from the medical staff is a big issue. That’s the major one that really needs to be addressed and I feel that there needs to be a lot of education done to overcome this barrier. I understand there are issues of hygiene and behavioural problems but I think through education in the universities we could tear down a lot of these barriers. (Social worker)"

## Discussion

Previous studies have identified interventions to improve the end-of-life care services for this population, highlighting the benefits of completing advanced planning directives [35-39] and emergency shelter-based end-of-life care [24,29], but have not focused on larger changes needed to the end-of-life care system. Our findings add to the literature by identifying systematic barriers to the end-of-life care system for homeless persons and articulating recommendations that have the potential to improve access and equity in this system for this population. Our study draws attention to the fact that poor access to the end-of-life care system is in part due to the fact that this system is in underdeveloped in Canada. Presently, the Canadian end-of-life care system is struggling to meet the demand its services due to the combination of an aging population and low levels of government investment in these services [44-46]. It is estimated that as few as 16% to 30% of people in Canada who die receive end-of-life care services [44,46]. It is apparent that a critical step of improving access to end-of-life care services for homeless populations is improving overall access to end-of-life care services through increased investment in this area.

Our findings also suggest that homeless populations encounter additional barriers to accessing the end-of-life care system that point to the need for changes in the organization and operating policies of this system. Across Canada, policymakers have increasingly championed death-at-home as the optimum outcomes of end-of-life care system and this desired outcome [46,47], which may inform how services are organized (e.g., home care or pay-for-service assisted living facilities). As a result, the end-of-life care system in some regions may not be developed in a way that accommodates services delivery to people who are homeless or marginally housed (e.g., living in substandard or transitional housing). A range of strategies, including hospital and non-profit hospice care, are be needed to ensure that those unable to access home care services or assisted living facilities have access to end-of-life care.

Furthermore, our research underscores the need for changes to operating policies within the end-of-life care system in order to better accommodate people who use drugs and/or experience mental illness. Our findings suggest that harm reduction strategies are perceived as a potential strategy to improve access to end-of-life care services for homeless persons because they remove one of the main barriers to hospice and end-of-life care services (i.e. anti-drug policies). Researchers have previously acknowledged that harm reduction strategies improve end-of-life care services delivery to homeless populations [24,29]. For example, Podymow et al. [24] found that integrating harm reduction approaches into a shelter-based hospice (i.e. permitting onsite alcohol use, providing sterile syringes, and permitting off-site illicit drug use) decreased overall healthcare costs by reducing the need for hospital and emergency medical services. More recently, researchers have observed that harm reduction services play a critical role in mediating access to end-of-life care services [29,30] and have called for the integration of supervised drug consumption services (e.g., permitting the use of pre-obtained illicit drugs under medical supervision) into end-of-life care services [29,30]. These strategies warrant careful consideration and further research is needed to identify the strategies or combination of strategies (e.g. syringe exchange and distribution, methadone maintenance treatment, medically-supervised drug consumption services, etc.) that best mediate access to the end-of-life care system for this population.

Our findings further emphasize the need for improvements in continuity of care and mental health and substance use training. The end-of-life care system may benefit from replicating interventions (e.g. intensive case management, integrated services, etc.) [48,49] shown to enhance continuity of care for homeless populations. In particular, patient navigators (i.e. trained peers or healthcare professionals who work with clients to help them overcome barriers to health care services [50]) might serve as important advocates for homeless persons as they try to navigate the end-of-life care system and help minimize the impact of discrimination and/or exclusionary policies [51]. Furthermore, formal links between end-of-life care and public health services (e.g. community committees) might enhance collaboration. Finally, our findings echo those previous studies by identifying a need for increased training in mental health and substance abuse among end-of-life care professionals [24,52].

### Limitations

This study has several limitations that should be taken into consideration. Our findings may have limited generalizability due to limited sample size. Also, several recommendations may have limited generalizability to settings that lack universal healthcare coverage. Participants were recruited largely from community settings and our findings only partly reflect changes necessary to improve mainstream end-of-life care services delivery to the homeless. Further research with mainstream end-of-life care providers is needed to get their perspective on end-of-life care services delivery to this population, and in particular why homeless populations are underserved by this system. Finally, this article is not based on interviews with homeless persons who had experience with the end-of-life care system. We acknowledge that further work is needed to explore this population's perceptions of and experiences with the end-of-life care system. We completed preliminary interviews (n = 5) with homeless persons receiving care at a low-threshold hospice but suspended this part of our study due to concerns about the quality of data (e.g., consistency of accounts, recall of events, etc.). Future research, and especially that aiming to identify ways to improve the end-of-life care system, could benefit from better drawing upon the experiences of homeless end-of-life care recipients.

## Conclusions

This article documented health and social services professionals views of the barriers that homeless persons face to accessing the end-of-life care system, as well as recommendations to improve access to this system for this population. While participants identified several barriers (i.e., insufficient availability of services, exclusionary operating policies, and poor continuity of care), they made key recommendations for improving the end-of-life care system for this population. In particular, participants identified the importance of structural changes to the delivery of end-of-life care services, emphasizing the importance of expanding services, integrating harm reduction approaches, and fostering partnerships with the public health system. These observations have the potential to be translated into policy and programmatic responses, notably the expansion of end-of-life care services, implementation of patient advocate programs, and adoption of harm reduction policies. For policymakers and health administrators concerned with increasing equity in the end-of-life care system for homeless populations, these recommendations present a possible way forward.

## Competing interests

The authors declare that they have no competing interests.

## Authors' contributions

MGY and RM designed the study and conducted data collection. All authors contributed to the data analysis. RM wrote the first draft of the manuscript. All authors contributed to the critical revision and approved the final content.

## Pre-publication history

The pre-publication history for this paper can be accessed here:

http://www.biomedcentral.com/1472-684X/11/14/prepub

## References

[B1] Laird G Homelessness in a growth economy: Canada’s 21st century paradox 2007 Sheldon Chumir Foundation for Ethics in Leadership, Calgary (AB)

[B2] Sermons MW White P State of homelessness in America 2011 National Alliance to End Homelessness, Washington (DC) 48

[B3] Stewart B US Congress McKinney Homeless Assistance Act. Pub L No. 100–77 1987

[B4] Hwang SW Homelessness and health CMAJ 2001 164 2 229 233 11332321 PMC80688

[B5] Snyder LD Eisner MD Obstructive lung disease among the urban homeless Chest 2004 125 1719 1725 10.1378/chest.125.5.1719 15136382

[B6] Jones CA Perera A Chow M Cardiovascular disease risk among the poor and homeless: what we know so far Curr Cardiol Rev 2009 5 69 77 10.2174/157340309787048086 20066152 PMC2803292

[B7] Hwang SW Bugeja AL Barriers to appropriate diabetes management among homeless people in Toronto CMAJ 2000 163 161 165 10934977 PMC80205

[B8] Fisher PJ Breakey WR The epidemiology of alcohol, drug, and mental disorders among homeless persons Am Psychol 1991 46 1115 1128 1772149 10.1037//0003-066x.46.11.1115

[B9] Grinman M Chiu S Rederlmeier DA Drug problems among homeless individuals in Toronto, Canada: prevalence, drugs of choice, and relation to health status BMC Public Health 2010 10 94 10.1186/1471-2458-10-94 20181248 PMC2841106

[B10] Goering P Tolomiczenko G Sheldon T Boydell K Wasylenki D Characteristics of persons who are homeless for the first time Psychiatr Serv 2002 53 1472 1474 10.1176/appi.ps.53.11.1472 12407279

[B11] Lehman AF Cordray DS Prevalence of alcohol, drug and mental disorders among the homeless: one more time Contemp Drug Probl 1993 20 355 383

[B12] Robertson MJ Clark RA Charlebois ED HIV seroprevalence among homeless and marginally housed adults in San Francisco Am J Pub Health 2004 94 1207 1217 10.2105/AJPH.94.7.1207 15226145 PMC1448423

[B13] Culhane DP Gollub E Kuhn R Schpaner M The co-occurrence of AIDS and homelessness: Results from the integration of administrative databases for AIDS surveillance and public shelter utilization in Philadelphia J Epidemiol Community Health 2001 55 515 520 10.1136/jech.55.7.515 11413184 PMC1731940

[B14] Nyamathi AM Dixon EL Robbins W Risk factors for Hepatitis C infection among homeless adults J Gen Intern Med 2002 17 134 143 10.1046/j.1525-1497.2002.10415.x 11841529 PMC1495011

[B15] Bagget TP O’Connell JJ Singer DE Rigotti NA The unmet health care needs of homeless adults: a national study Am J Pub Health 2010 100 1326 1333 10.2105/AJPH.2009.180109 20466953 PMC2882397

[B16] Gelberg L Gallagher TC Andersen RM Koegel P Competing priorities as barrier to medical care among homeless adults in Los Angeles Am J Public Health 1997 87 217 220 10.2105/AJPH.87.2.217 9103100 PMC1380797

[B17] Wen C Hudak PL Hwang SW Homeless people’s perceptions of welcomeness and unwelcomeness in healthcare encounters J Gen Intern Med 2007 22 1011 1017 10.1007/s11606-007-0183-7 17415619 PMC2219712

[B18] Martins CM Experiences of homeless people in the health care delivery system: a descriptive phenomenological study Public Health Nurs 2008 25 420 430 10.1111/j.1525-1446.2008.00726.x 18816359

[B19] Hwang SW Mortality among men using homeless shelters in Toronto, Ontario JAMA 2000 283 2152 2157 10.1001/jama.283.16.2152 10791509

[B20] Cheung AM Hwang SW Risk of death among homeless women: a cohort study and review of the literature CMAJ 2004 170 1243 1247 10.1503/cmaj.1031167 15078846 PMC385354

[B21] Hwang SW Wilkins R Tjepkema M O'Campo P Dunn JR Mortality among residents of shelters, rooming houses, and hostels in Canada: 11 year follow-up study BMJ 2009 339 1065 1070 10.1136/bmj.b4036 PMC2767481 19858533

[B22] Morrison DS Homelessness as an independent risk factor for mortality: results for a retrospective cohort study Int J Epidemiol 2009 38 877 883 10.1093/ije/dyp160 19304988

[B23] Barrow SM Herman DB Cordova P Struening EL Mortality among homeless shelter residents in New York City Am J Pub Health 1999 89 529 534 10.2105/AJPH.89.4.529 10191796 PMC1508869

[B24] Podymow T Turnbull J Coyle D Shelter-based palliative care for the homeless terminally ill Palliat Med 2006 20 81 86 10.1191/0269216306pm1103oa 16613403

[B25] Cagle J Weathering the storm: Palliative care for the elderly homeless J Hous Elderly 2009 23 29 46 10.1080/02763890802664588

[B26] Sermonds MW Henry M Demographics of homelessness series: The rising elderly population 2010 National Alliance to End Homelessness, Washington (DC) Apr. 8p

[B27] Steriopoulos V Herrman N Old and homeless: A review and survey of older adults who use shelters in an urban setting Can J Psychiatry 2003 48 374 380 12894611 10.1177/070674370304800603

[B28] Garibaldi B Conde-Martel A O’Toole TP Self-reported comorbidities, perceived needs, and sources for usual care for older and younger homeless adults J Gen Intern Med 2005 20 726 730 10.1111/j.1525-1497.2005.0142.x 16050882 PMC1490194

[B29] McNeil R Guirguis-Younger M Illicit drug use as a challenge to the delivery of end-of-life care services to homeless persons who use illicit drugs: Perceptions of health and social care professionals Palliat Med 2012 26 350 359 10.1177/0269216311402713 21464120

[B30] McNeil R Guirguis-Younger M Dilley LB Aubry TD Turnbull J Hwang SW Harm reduction services as a point-of-entry to and source of end-of-life care and support for homeless and marginally housed persons who use alcohol and/or illicit drugs: a qualitative analysis BMC Pub Health 2012 12 312 10.1186/1471-2458-12-312 22545586 PMC3355019

[B31] Williams AM Crooks VA Whitfield K Tracking the evolution of hospice palliative care in Canada: A comparative case study analysis of seven provinces BMC Health Serv Res 2010 10 147 10.1186/1472-6963-10-147 20515491 PMC2898768

[B32] Wilson DM Truman CD Thomas R The rapidly changing location of death in Canada, 1994–2004 Soc Sci Med 2009 68 1762 1768 10.1016/j.socscimed.2009.03.006 19342137

[B33] Hwang SW O’Connell JJ Lebow JM Health care utilization among homeless adults prior to death J Health Care Poor Underserved 2001 12 50 58 10.1353/hpu.2010.0595 11217228

[B34] Hanzlick R Parrish RG Death among the homeless Fulton County, GA, 1988–1990 Public Health Rep 1993 108 488 491 8341785 PMC1403414

[B35] Song J Bartels DM Ratner ER Alderton L Hudson B Ahluwalia JS Dying on the streets: Homeless persons’ concerns and desires about end of life care J Gen Inter Med 2007 22 435 441 10.1007/s11606-006-0046-7 PMC1829423 17372789

[B36] Song J Ratner ER Bartels DM Alderton L Hudson B Ahluwalia JS Experiences with and attitudes toward death and dying among homeless persons J Gen Intern Med 2007 22 427 434 10.1007/s11606-006-0045-8 17372788 PMC1829422

[B37] Tanzian AJ Neal MT O’Neil JA Attitudes, experiences and beliefs affecting end-of-life decision-making among homeless individuals J Pall Med 2005 8 36 48 10.1089/jpm.2005.8.36 15662172

[B38] Song J Wall MM Ratner ER Engaging homeless persons in end of life preparations J Gen Intern Med 2008 23 2031 2045 10.1007/s11606-008-0771-1 18800207 PMC2596520

[B39] Song J Ratner ER Wall MM Effect of an end-of-life planning intervention on the completion of advance directives in homeless persons: A randomized trial Ann Intern Med 2010 153 1 38 20643989 10.7326/0003-4819-153-2-201007200-00003

[B40] Guirguis-Younger M McNeil R Runnels V Learning and knowledge-integration strategies of nurses and client care workers serving homeless persons Can J Nurs Res 2009 41 20 34 19650511

[B41] Hwang SW Ueng JM Chiu S Universal health insurance and health care access for homeless persons Am J Pub Health 2010 100 1454 1461 10.2105/AJPH.2009.182022 20558789 PMC2901287

[B42] Glaser B Strauss A The discovery of grounded theory: Strategies for qualitative research 1967 Aldine, New York

[B43] Strauss A Corbin J Basics of qualitative research: Grounded theory procedures and techniques 1990 Sage, Thousand Oaks, CA

[B44] Policy brief on palliative care Quality end-of-life care? It depends on where you live…and where you die 2010 Canadian Hospice and Palliative Care Association, Ottawa (ON) 12

[B45] Williams AM Eby JA Stajduhar K Canada’s Compassionate Care Benefit: Is it an adequate public health response to addressing the issue of caregiver burden in end-of-life care? BMC Pub Health 2011 11 335 10.1186/1471-2458-11-335 21592383 PMC3123207

[B46] Carstairs S Raising the bar: A roadmap for the future of palliative care in Canada 2010 The Senate of Canada, Ottawa (ON)

[B47] Decision-making at end of life 2007 The College of Physicians and Surgeons of Ontario, Ottawa (ON) 8

[B48] Ploeg J Hayward L Woodward C Johnston R A case study of a Canadian homelessness intervention programme for elderly people Health Soc Care Community 2008 16 593 605 10.1111/j.1365-2524.2008.00783.x 18371167

[B49] Lehman AF Dixon LD Kernan E DeForge BR Postrado LT A randomized trial of assertive community treatment for homeless persons with severe mental illness Arch Gen Psychiatry 1997 54 1038 1043 10.1001/archpsyc.1997.01830230076011 9366661

[B50] Rosenheck R Morrissey J Lam J Service integration, access to services, and housing outcomes in a program for homeless persons with severe mental illness Am J Pub Health 1998 88 1610 1615 10.2105/AJPH.88.11.1610 9807525 PMC1508580

[B51] Freeman HP Patient navigation: A community based strategy to reduce cancer disparities J Urb Health 2006 83 139 141 10.1007/s11524-006-9030-0 PMC2527166 16736361

[B52] McCasland L Providing hospice and palliative care to the seriously and persistently mentally ill J Hosp Palliat Nurs 2007 9 314 315 10.1097/01.NJH.0000299329.47042.ac

